# Application properties and biosafety enhancement in saliva collection: optimization of a non-blowing medical device

**DOI:** 10.1186/s12903-026-08834-1

**Published:** 2026-06-11

**Authors:** Dong Zhao, Jinrong Zhao, Yijing Li, Jihu Sun

**Affiliations:** 1 Applied Research Institute of Life Sciences, Xi’an International University, Xi’an, Shaanxi China; 2 School of Medicine, Xi’an International University, 18 Yudou Road, Xi’an, Shaanxi 710077 China

**Keywords:** Enclosed cavity, Medical device, Saliva, Non-blowing collection, Applications

## Abstract

**Background:**

Saliva stands out among diverse biological samples, offering readily available DNA samples through non-invasive collection methods. This open sampling method, which uses repetitive blowing actions for saliva collection with increased safety risks, poses a matter of concern.

**Methods:**

By employing the non-blowing method, this study designed a closed-chamber medical device for saliva collection. Compared with repetitive collection in commercial devices (Class I medical devices), its sampling efficiency was assessed with quantitative saliva. After being stored in this device for six months, DNA was extracted and amplified integrating PCR with high-resolution melt (HRM) via a detection kit. Twelve-month saliva samples underwent a limit test under the minimum limit DNA template. The applications for concordance among paired blood–saliva samples was further assessed in microarrays.

**Results:**

Non-blowing saliva collection offered closed-chamber biosecurity and its collection efficiency was comparable to that of commercial devices (Z = -0.778, *P* = 0.477). After six months of storage at room temperature, DNA extraction and quantification of saliva showed that DNA yield from the non-blowing saliva collection was also comparable to that of the commercial device (Z = -1.886, *P* = 0.064). Six- and 12-month saliva samples stored in this non-blowing device solution at room temperature maintained a reliable DNA quantity and quality for HRM, comparable to those of commercial devices with repetitive blowing-type. In the limit test, PCR-HRM demonstrated that stable extracted-DNA was successfully genotyped, and the peaks-valleys also tended to be consistent between the two devices. For paired blood–saliva samples, quality-control parameters in microarrays were within the feasible quality limits.

**Conclusions:**

Non-blowing type saliva collection device has good application performance in both PCR-HRM and microarray. This study presents a new medical device designed to provide healthcare professionals with a biosecure and practical solution for saliva sample acquisition.

**Supplementary Information:**

The online version contains supplementary material available at 10.1186/s12903-026-08834-1.

## Introduction

DNA testing is a pivotal tool for clinical disease screening, diagnosis, and genetic analysis, with critical applications in personalized medicine. Available DNA can be extracted from diverse biological samples, including blood, buccal swabs, saliva [1, 2], and other sources.

Blood samples are widely used in diagnostic laboratories because of their high DNA quality and yield. However, their invasive collection requires the involvement of healthcare professionals, and long-term storage or transport often necessitates dry ice, ice packs, or cryogenic equipment. Although smaller amounts of DNA can be extracted from buccal swabs and hair roots, these sources remain less efficient. In contrast, ‌saliva‌ offers a ‌noninvasive alternative‌ with distinct advantages. It eliminates the need for medical personnel, provides high DNA yield (average 35.5 µg per 2 mL of saliva [3]), and supports cost-effective room-temperature storage and transport. Saliva-derived DNA originates from leukocytes and oral epithelial cells. The content of bacterial DNA is also sparse, with bacteria at approximately 10^9^-10^10^ CFU/mL in saliva samples [4], which is notably superior to that of buccal swabs [5]. These properties position saliva as a reliable alternative source of genomic DNA (gDNA) for genetic testing, particularly in PCR-based assays.

A variety of saliva collectors have been commercialized and are primarily categorized into types such as ‌passive drool and mouthwash protocols. The mouthwash protocol is not widely promoted because of its comparatively low DNA yield and quality and excessive volume after the preservation of liquid gargling [6]. It is also unfavorable to subsequent suitability and operation for advanced technologies. In particular, ‌passive drool collection commonly involves repetitive blowing. Owing to their reliable strengths of simplicity and psychological acceptance, these repetitive blowing-type devices are also widely adopted in the field of direct-to-consumer genetic testing through mailing a spit kit [7]. However, droplet transmission during saliva collection poses a risk of the spread of pathogens, including COVID-19. This concern has driven the rapid adoption of collection with spit saliva restriction devices during the COVID-19 pandemic [8]. Despite these challenges, saliva remains a viable biosample for the detection of infectious diseases, e.g., COVID-19. Nevertheless, healthcare professionals must collect such samples with strict precautions.

Notably, this collection method requires performing repetitive blowing actions. This unsightly process generates droplets and aerosols that increase biosafety risks. The expelled saliva frequently contains foreign matter such as foam and viscous mucus, which interferes with downstream processing steps. To address these limitations, this study aims to redesign existing repetitive blowing devices and develop a non-blowing saliva collection device, hereafter termed the new device. To evaluate the suitability of DNA derived from the new device, we also compared its performance in PCR-based assays with that of a commercially available blowing-type device (hereafter termed the commercial device), which served as the reference standard.

## Methods

### Functional design

**Fig. 1:**
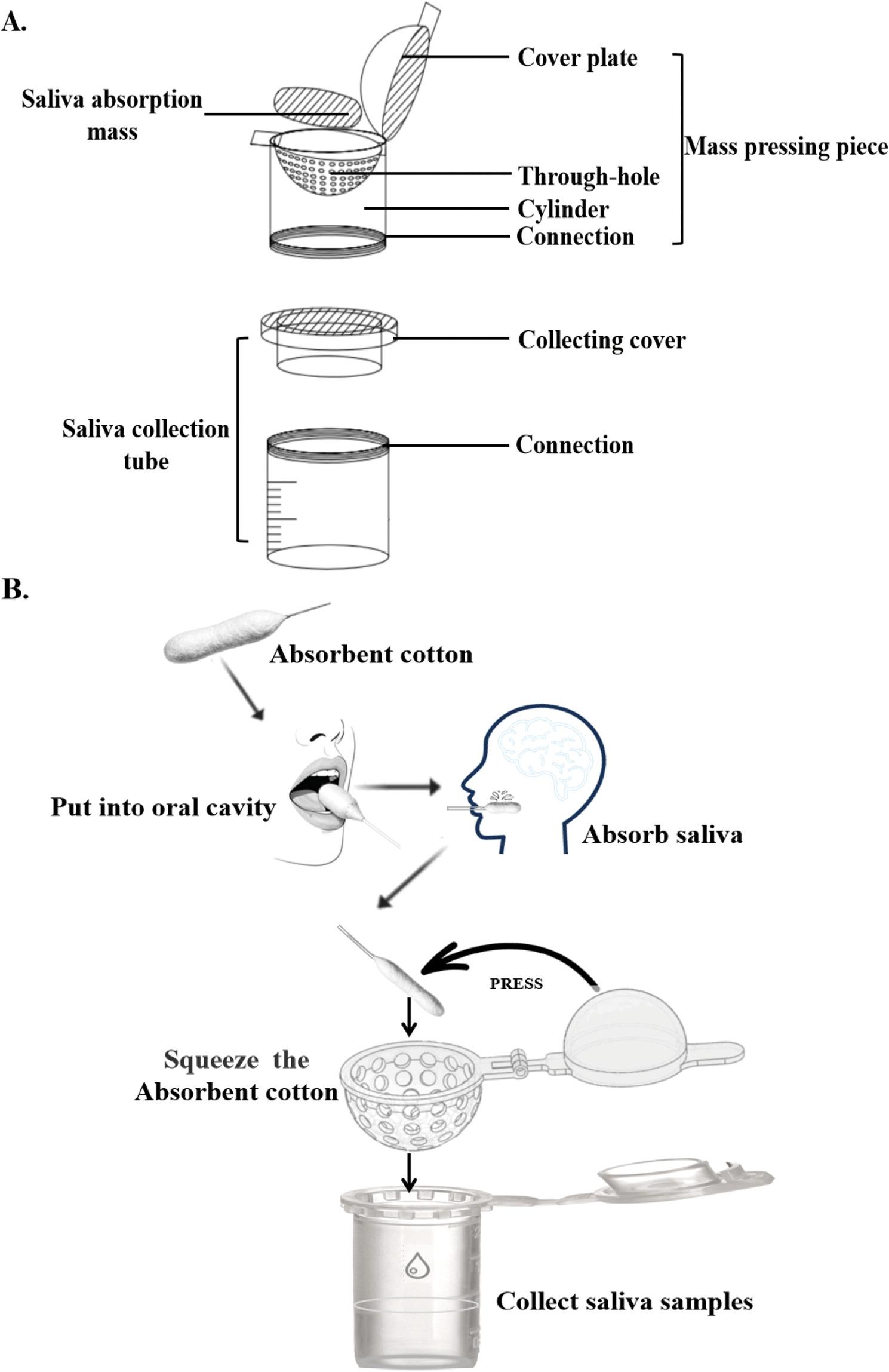
Schematic diagram (**A**) and operation flowchart (**B**) of the non-blowing saliva collection device‌. Taking absorbent cotton as a model material

To mitigate biohazard risks associated with droplet and aerosol transmission during saliva collection, this study designed an innovative closed-system saliva sampling apparatus engineered with biosafety architecture. The new device integrates three functionally optimized components enabling non-blowing collection: (a) a saliva absorption mass made of medical-grade absorbent cotton, in the oral cavity, physiologically stimulates salivary glands for low-stress secretion while actively absorbing saliva, (b) a mass pressing piece, wherein a cover plate on the upper-outer wall is flexibly linked with the principal part at the bottom, and through-hole component attached to the plate body filters out foreign matter equally, and (c) a calibrated saliva collection tube detachably connected to the opening at the bottom of the cylinder, allowing for the collection of saliva in sufficient quantities as needed. The structure diagram of the new medical device is shown in Fig. 1(A).

During practical saliva collection, a strip of absorbent cotton tied with a string is employed. Grasp the string and put the absorbent cotton into the ‌closed oral cavity. No spitting or blowing actions are required from the subject, as the cotton automatically absorbs saliva. Subsequently, transfer the saliva-saturated absorbent cotton into the detachable plate body, then press down on the cover plate. Saliva is squeezed out of the absorbent cotton and simultaneously collected in the saliva collection tube. The step-by-step operation flowchart is displayed in Fig. 1(B).

### Sample collection and quantification

This study was conducted following the Declaration of Helsinki and was approved by the Life Science and Medical Ethics Committee of Xi’an International University (approval no. XAIULL-2021 007). All saliva collection volunteers (Nos. 001–010) were over the age of 18 and had provided written informed consent to participate in both the application and evaluation of the new saliva collector.

To validate the present device, commercial devices classified as medical devices were used as experimental controls. For commercial devices, the protocols employed repetitive blowing-type method to obtain a sufficient sample volume. After rinsing their mouths with water, all participants were instructed to avoid eating, drinking, smoking, or chewing gum for 30 min.

After training, all volunteers (aged > 18 years) exhibited independent competence in the operation of both devices. On the first day, half of the participants were randomly selected to provide saliva samples via the commercial device, whereas the other half were assigned to use the new device. The group assignments were reversed on the following day. Samples were collected with both devices simultaneously within 5 min. The collection volume was recorded after foreign matter was removed.

### DNA extraction and quantification

The samples collected from the commercial device were stored for 6 months (at room temperature) according to the manufacturer’s instructions. For the new device, saliva samples were mixed with preservation solution at 1:1 ratio (v/v). The preservation solution consisted of the following components: 200 mmol/L NaCl, 20 mmol/L Tris·Cl (pH 8.0), 20 mmol/L EDTA (pH 8.0), 1% sodium dodecyl sulfate (SDS), and 0.2 mg/mL proteinase K. The samples were subsequently stored individually at room temperature (~ 25 °C) and −80 °C up to 6 months. All samples (1000 µL) subsequently underwent DNA extraction via the classical phenol‒chloroform method [9]. The extracted DNA was finally dissolved in 50 µL of sterilized purified water. The DNA concentration and A_260_/A_280_ ratio were determined via Nanodrop One spectrophotometric analysis.

### Application testing of DNA

The extracted DNA was analyzed by integrating PCR with HRM curve analysis to confirm its usability for genetic research. DNA derived from the new device (stored at room temperature) and the commercial device was used as the PCR template. Under optimum conditions with 70 ng of DNA per reaction, DNA samples were amplified using a detection kit for the psoriasis susceptibility genetic variant POU5F1 rs1265159 (developed by the Engineering Research Center of Personalized Anti-Aging Health Product Development and Transformation, Shaanxi). The PCR-based SNP genotyping results from each collection method were validated and analyzed. On the QuantStudio 5 Real-Time (Applied Biosystems, USA) platform, the PCR system was set up as follows: the PCR mixture (25 µL total volume) contained 12.5 µL of 2×ChamQ master mix, 0.5 µL each of 10 µM forward and reverse primers, 1.25 µL of 20×EvaGreen, and 70 ng of DNA template, with dH₂O for the final volume adjustment. The amplification conditions were as follows: predenaturation at 94 °C for 180 s and 40 cycles of 95 °C for 20 s, 68 °C for 30 s (fluorescence collection), and 72 °C for 30 s. The melting curve analysis conditions were: 65 °C for 1 min, then 0.05 °C/s ramp to 95 °C with fluorescence collection. Genotype-confirmed standard controls (AG and GG) were included in all PCR-HRM runs. The melting temperature (Tm) was automatically generated and documented by the instrument’s integrated analytical software suite.

At the study time points, a limit test was conducted to assess DNA reliability in saliva samples stored for up to 12 months. Under these experimental conditions, the samples derived from the commercial device served as the control. The rs1265159 point mutation was genotyped with 70 ng of DNA template per reaction. For the new device, rs1265159 genotyping was performed at a minimum DNA threshold of 21 ng per reaction. All the PCR-HRM results were analyzed systematically. The amplified products were assessed via 2% agarose gel electrophoresis.

We also evaluated its application in microarrays. DNA derived from the paired blood-saliva samples was processed using the same protocol. Blood samples, which were provided by ten volunteers (Nos. 001–010) from the representative lineage, served as a standard and were compared with paired saliva samples. Saliva samples were stored in solution at room temperature for 6 months, whereas blood samples were stored at -80 ℃. Following standard protocols, genotyping was performed using the high-throughput Infinium Asian Screening Array (ASA).

### Data analysis

In the descriptive statistics, continuous variables (Saliva volume, DNA concentration, and A_260_/A_280_ ratio) are presented as median and interquartile ranges (IQRs). Based on the distribution characteristics, the non-parametric Wilcoxon Signed-Rank test was employed for comparisons of these continuous variables between groups. All statistical tests were two-sided, and a *P* value <0.05 was considered indicative of statistical significance. All analyses were performed using IBM SPSS Statistics software (version 24).

## Results

### Saliva collection efficiency

Under supervised collection, all participants successfully provided saliva samples. The median saliva volume in commercial device and new device groups was 2.35 (IQR: 2.27-2.60) and 2.30 (IQR: 2.18-2.53) mL, respectively. Compared with the commercial device, the new device demonstrated a nonsignificant (*Z = -0.778*,* P* = 0.477) increase in average saliva collection volume (2.34 ± 0.20 mL). The results are shown in Table 1.

**Table 1:** Performance characteristics of DNA extracted from saliva derived from the two devices

Parameter‌		Commercial device (Ref)	New device	‌Statistical Comparison (Wilcoxon Signed-Rank test)‌
‌Saliva Volume (mL)‌				
Mean ± SD		2.40 ± 0.19	2.34 ± 0.20	*Z = -0.778*,* P* = 0.477
Median (IQR)		2.35 (2.27-2.60)	2.30 (2.18-2.53)	
‌DNA Concentration (ng/µL)‌				
After 6-month RT storage	Mean ± SD	488.2 ± 61.7	467.9 ± 66.1	*Z = -1.886*,* P* = 0.064
	Median (IQR)	486.3(444.1-508.0)	462.8(422.9-495.8)	
After 6-month − 80 °C storage	Mean ± SD	—	249.9 ± 27.1	*Z = -2.803*,* P* = 0.002 ^†^
	Median (IQR)		250.7(241.2-256.8)	
A260/A280 ratio				
After 6-month RT storage	Mean ± SD	1.63 ± 0.09	1.65 ± 0.13	*Z = 0.051*,* P* = 0.973
	Median (IQR)	1.60(1.55-1.68)	1.61(1.54-1.78)	
After 6-month − 80 °C storage	Mean ± SD	—	1.70 ± 0.09	*Z = 1.786*,* P* = 0.078
	Median (IQR)		1.69(1.65-1.77)	

*RT* room temperature

Data are presented as ‌mean ± standard deviation‌ and ‌median (interquartile range, IQR)‌ to reflect both central tendency and distribution

^†^ Significant difference compared to Commercial device (reference group)

Comparisons were performed using the Wilcoxon Signed-Rank test‌; statistical significance was set at *P* < 0.05

### DNA acquisition assessment

We extracted DNA from each of the samples using the same phenol‒chloroform method and subsequently measured concentration and A_260_/A_280_ ratio. Table 1 presents the concentration and quality of the DNA extracted from the saliva after 6-month storage. The detectable levels of gDNA in each sample were derived from the commercial device and new device (stored at room temperature and -80 °C) with average concentrations of 488.2 ± 61.7 ng/µL, 467.9 ± 66.1 ng/µL and 249.9 ± 27.1 ng/µL, respectively. The A_260_/A_280_ ratio of the DNA ranged from 1.5 to 1.9. Compared with those of the commercial device, the DNA concentrations of the new device were not significantly different at room temperature (*Z = -1.886*,* P* = 0.064). The storage condition of -80 °C significantly reduced the DNA yield of the new device (*Z = -2.803*,* P* = 0.002). Similarly, the A_260_/A_280_ ratio of DNA extracted using the new device was comparable to that obtained with commercial device at room temperature (*Z = 0.051*,* P* = 0.973). Although the mean A_260_/A_280_ ratio of DNA from the new device increased at -80 °C, this elevation did not reach statistical significance (*Z = 1.786*,* P* = 0.078). These findings are comprehensively summarized in Table 1.

### Application test of DNA

**Fig. 2:**
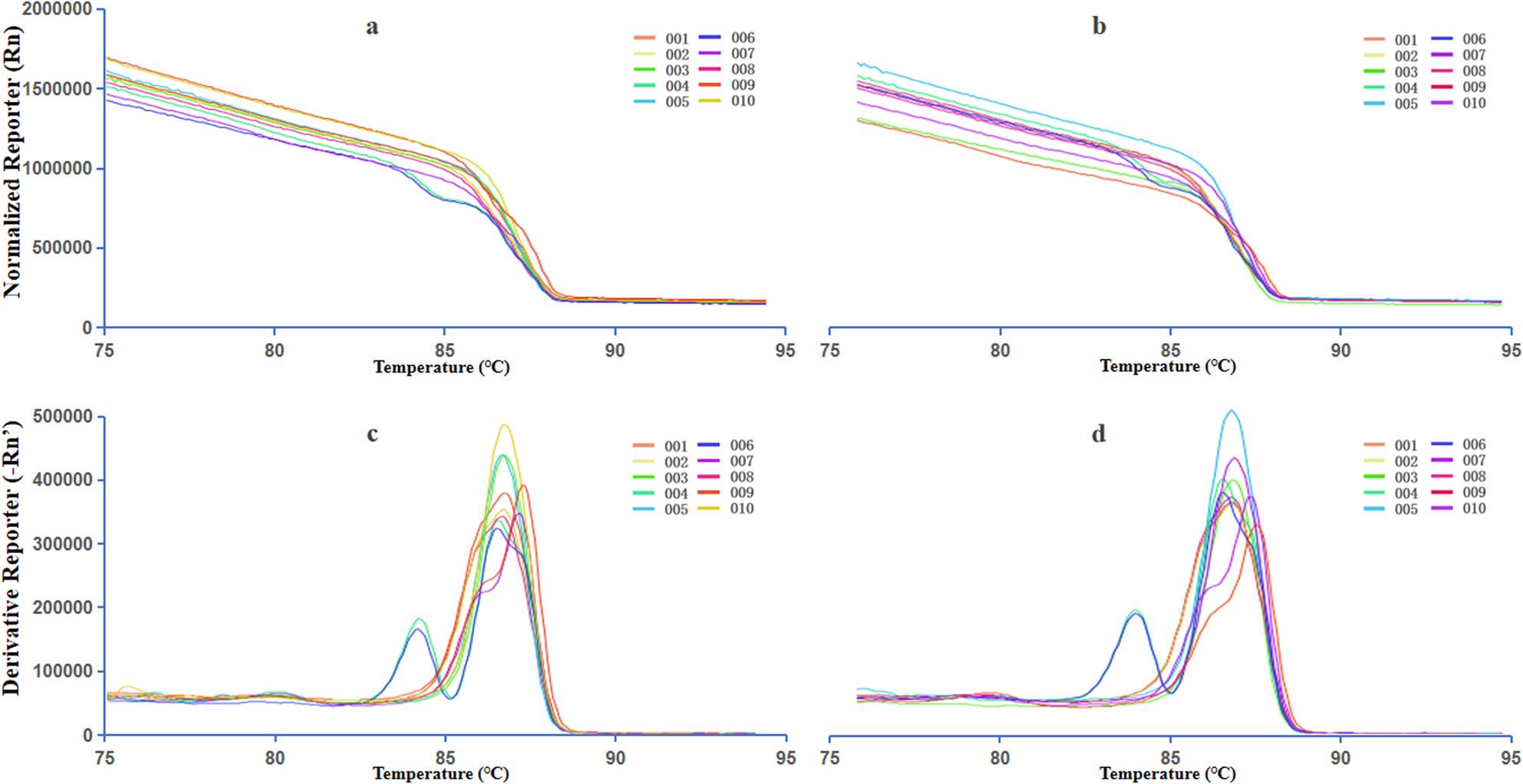
PCR-HRM analysis results for saliva samples (Nos. 001–010) following 6 months of storage. Images of normalized and derivative reporters generated from the new device (a, c) and commercial device (b, d) with 70 ng per reaction templates. Standard reference materials included Sample 004 (heterozygous, AG) and Sample 010 (homozygous, GG)

**Fig. 3:**
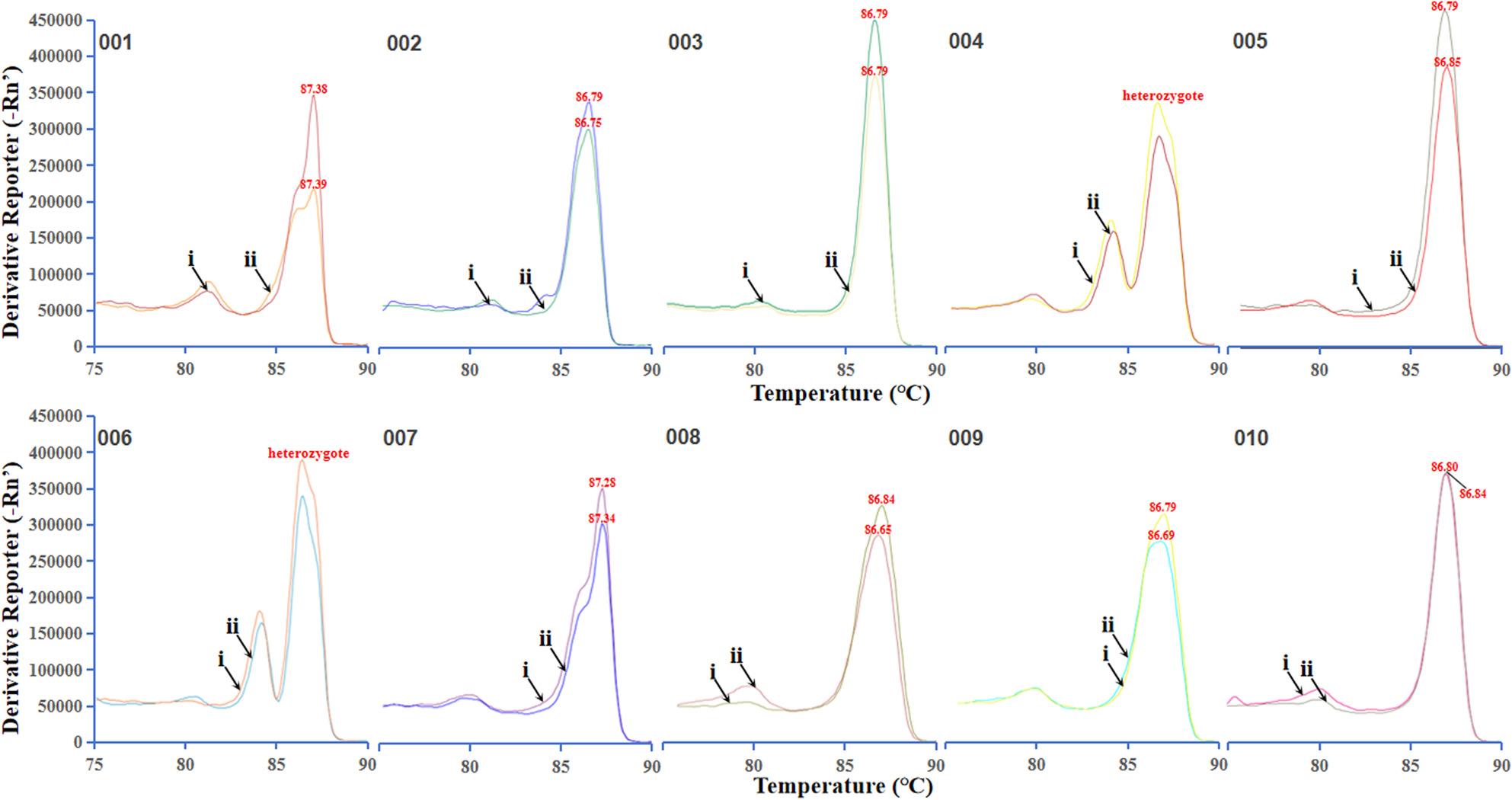
Results of PCR-HRM for saliva samples (Nos. 001–010) stored for up to 12 months. Images of the derivative reporter after PCR-HRM with various devices (limit test): (ⅰ) Commercial device, detection kit was used for PCR-HRM analysis with 70 ng of DNA per reaction; (ⅱ) New device, point mutation was identified with a minimum limit of 21 ng of DNA per reaction. Standard reference materials included Sample 004 (heterozygous, AG) and Sample 010 (homozygous, GG)

Saliva samples collected by the new device (room temperature storage) were used for DNA extraction and rs1265159 genotyping. PCR-HRM analysis was performed on DNA extracted from 6-month stored saliva. All the samples were correctly genotyped, as were those processed with the commercial device. This finding was analyzed in conjunction with limit test results from samples stored for 12 months. The normalized reports and derivative reports revealed that the ΔTm value was ≤ 0.19 °C between the two devices, and the peaks-valleys also had a consistent trend with a smooth line, indicating reliable genotyping (Figs. 2 and 3).

The HRM results demonstrated that stable extracted-DNA was successfully genotyped within the limiting condition of 21 ng template per reaction, even when saliva samples were stored in the new device for up to 12 months at room temperature. The peaks-valleys from the normalized reports and derivative reports tended to have the same trend (Fig. 3, Additional file 1). All reports revealed that the values of Rn and -Rn’ varied with time and progress, indicating excellent ability of two devices to provide stable and high-quality samples. Under both 70 ng and 21 ng DNA template conditions, all homozygous (GG) samples exhibited higher Tm values compared with the heterozygous (AG) samples (004 and 006) (Additional file 2 Table 1). The size of the POU5F1 regions amplified by PCR was approximately 177 bp. The electrophoresis results did not detect any obvious effect of the DNA template derived from either device (see Additional file 3).

Considering the accessibility of the ASA array, comparative analysis was conducted for the blood–saliva sample pairs, and the quality-control parameters were compared with those obtained from the blood samples. 100% identical rs1265159 genotypes were characterized from the results of the ASA array and first-generation sequencing. The chip detection results between the two-type devices demonstrated > 99.8% concordance across 741,988 SNPs. Moreover, the call rate from the ASA array exceeded 99.0%, which reached high sensitivity.

## Discussion

This study provided a novel device for saliva collection via a non-blowing method. From absorbing saliva to filtration of foreign matter, the three principal parts created an enclosed space for safety, that is, effectively eliminating droplet and aerosol transmission risks. The successful application of HRM and microarrays conclusively established saliva collection via this method as a viable alternative for acquiring ‌derived gDNA from a stable source.

Saliva-derived DNA collection offers a non-invasive method for analysis, serving as a potential alternative to blood sampling for molecular testing, particularly in population-scale screening [10]. However, repetitive blowing saliva collection inherently operates as open sampling. When an infected person is identified as a sampled individual, all processes can be placed into biohazard zones. In this process of repeatedly blowing saliva, the droplets and aerosols, classified as a continuum of particle sizes of IRPs (infectious respiratory particles), create persistent challenges for infection prevention [11]. To address this, we specifically developed a closed-chamber collection device for saliva absorption. Within the closed oral cavity, the saliva absorption mass, a core component, achieved saliva fluid absorption to prevent unfavorable consequences from droplets and aerosols.

Salivary biomarkers, particularly DNA and extracellular components, provide stable diagnostic material for oral diseases like caries and oral cancer [12, 13, 14]. Salivaomics integrates multi-omic approaches (genomics, proteomics, metabolomics) to identify disease-specific molecular signatures in oral pathology [15]. Crucially, non-invasive sampling devices enable collection without compromising analytical validity, facilitating reliable detection of pathogens (e.g., *S. mutans* genotypes in caries), and cancer biomarkers (oral squamous cell carcinoma) [13, 16]. This supports robust epidemiological research and clinical diagnostics [12, 15].

We found comparable saliva collection volumes between the repetitive blowing-type and non-blowing methods, with both devices achieving a minimum volume within 5 min. In this study, the concentration of DNA derived from the new device (467.9 ± 66.1 ng/µL) was not significantly different from that derived from the commercial device (488.2 ± 61.7 ng/µL) at room temperature for 6 months (*Z = -1.886*,* P* = 0.064), confirming that the total DNA yields of the two devices were comparable. After equivalent conversion, the total DNA yields were approximately 23.3 µg and 24.4 µg, respectively, which exceeded the average yield [3].

It should be mentioned that saliva samples from this new device should be kept at room temperature and avoid freezing. It was reported that even single freezing significantly could reduce DNA yield (> 25%) in blood specimens [17]. For saliva, Nemoda et al. systematically investigated whether archived samples subjected to multiple freeze-thaw cycles yield DNA of sufficient quantity and quality for genetic analyses. They found freeze-thaw cycles adversely affected both DNA concentration and quality, with frozen samples exhibiting higher A_260_/A_280_ ratios [18]. These adverse conditions likely induced DNA damage [19] or enhanced decomposition of cellular material, increasing large-molecule contaminants [18]. Similar to the findings of other studies, our study observed that DNA extracted from saliva stored at -80℃ for 6 months exhibited significantly reduced concentration (*Z =* -2.803, *P* = 0.002). This may be attributed to the lack of cryoprotectants in the new device. Interestingly, even with relatively low DNA concentrations, successful amplification of human genetic polymorphisms [18, 20] and analysis of salivary biomarkers [21, 22] remain achievable in cryopreserved saliva specimens. Further investigation into optimized cryoprotectants or DNA stabilizers may facilitate the development of more robust saliva cryopreservation methods, making this device more widely applicable.

PCR amplification combined with HRM has been widely applied to SNP genotyping with high sensitivity and specificity. As demonstrated by Liew et al. in their detailed study of single-nucleotide polymorphisms using small-amplicon high-resolution melting analysis, heterozygotes were easily identified by altered melting curve shapes (e.g., bimodal peaks) due to heteroduplex formation. Conversely, homozygotes were genotyped by Tm differences of 0.8–1.4 °C [23, 24]. In our genotyping of rs1265159, homozygotes from one particular PCR product served as known homozygous genotypes GG. For each product, all homoduplex amplifications were easily identified under AA or GG as a reference genotype. Our results aligned with these principles: all derivative reports confirmed that heterozygotes (e.g., samples 004 and 006) with bimodal peaks were clearly differentiated from homozygotes. For the rs1265159 variant (binary combinations of bases, G > A), homozygotes (GG) consistently exhibited higher Tm values than heterozygotes (AG), as validated by our instrument’s integrated analytical software (Additional File 2 Table 1). Considering the stability of commercial devices storing samples for up to 12 months, we also conducted PCR amplification and HRM on the device within the limiting 21 ng template per reaction under such conditions. Integrated with PCR-HRM data from 6-month stored saliva, all the normalized and derivative reports revealed that heterozygotes (Fig. 3 004 and 006) with a bimodal peak were clearly differentiated from homozygotes. As expected, the peaks-valleys also tended to have the same trend additionally (Figs. 2 and 3), and the gels of PCR products had intact morphological structures with high molecular stability. Saliva samples stored in both devices at room temperature for 12 months were stable for genotype detection (Additional file 2 Table 1). Predictably, this saliva sample will provide key logistical benefits in sample shipping and storage at room temperature for large-scale epidemiological studies [25].

Application studies were further conducted on both HRM and microarray platforms, confirming the adaptability of saliva derived from the new device for various scenarios. Our results demonstrate that saliva collected via the new device yields gDNA quality comparable to that of the commercial device, supporting its utility in complex genomic analyses. The extracted gDNA exhibited excellent quality, despite contamination probably characterized by the presence of nonhuman DNA sequences (e.g., bacterial genomes) at varying percentages [1, 26]. Consequently, to circumvent the confounding effects of contamination, we designated the blood sample as our current standard reference for microarrays. This approach specifically excluded saliva derived from the commercial device while meeting a strict test protocol. In clinic diagnostic, blood analysis is regarded as the gold standard for monitoring analytes. Using blood as an independent reference circumvents possible non-human contamination while providing an excellent quality benchmark for our research.

The saliva samples collected by the new device were paired with blood, which yielded high-quality data through ASA array analysis performed on these matched biospecimens. Variants, including the ASA array with call rate > 99.0%, were identified. These array results indicated that the genotyping accuracy was comparable to that of most commercial SNP arrays [27]. Benefiting from its easy-to-use sampling design, the new device has successfully completed a series of independent saliva collection tests and has entered commercial trial stages. To preserve the objectivity of the evaluation, independent Infinium Asian Screening Array (ASA) detection and analysis were conducted on samples collected by the new device, with all procedures carried out by third party testing agencies. The test results of the latest 28 samples demonstrate that the call rate of the ASA array exceeds 98.2%, achieving a high level of sensitivity (Additional file 2 Table 2). On the basis of the above findings, a quality assessment of the data demonstrated that the parameters from the ASA array and HRM matched the standard practices in the field. Extending these findings to DNA sequencing, the new device showed potential to overcome limitations associated with conventional saliva collection methods. The consistency in DNA concentration and purity, coupled with its performance in HRM and microarray assays, ensured that saliva samples met quality control requirements and reproducibility for DNA sequencing platforms [28, 29]. This finding indicated that the standardized collection method for saliva samples on the new device likely demonstrated its compatibility with these platforms. Future work should focus on direct comparisons with blood-derived DNA in whole-genome. By addressing these aspects, the device may establish a new benchmark for non-invasive sampling in large-scale genomic studies.

Although the samples isolated from the device provided a high yield and high quality of DNA, our study had certain limitations. Firstly, the new device is being applied for filing with the administration as a Class I medical device. Subsequent trials in larger samples will be essential for its future clinical usage. Secondly‌, as a comparative study with the commercial device, saliva stored in the new device at room temperature for up to 12 months still confirmed the effectiveness of PCR-HRM approaches for genotyping. This performance precisely underscored the device’s practical utility, eliminating reliance on specialized cold storage infrastructure. In the future longer storage durations still need to be tested. Finally, the closed, non-blowing design of new device inherently minimized the potential for droplet and aerosol generation compared to traditional open collection systems. Validating these theoretical advantages demands direct measurement of droplet and aerosol emissions during sample collection. This measurement could be accurately conducted in future studies using particle counters or comparable specialized instruments.

## Conclusion

This exploratory study developed, for the first time, a closed-chamber collection device for saliva that enabled adequate sample acquisition through a non-blowing type while preventing droplet and aerosol transmission. Furthermore, non-blowing type saliva collection device has good application performance in both PCR-HRM and microarray. While the small sample size limits the generalizability of these findings, this device, currently in the process of being commercialized, offers healthcare professionals a potential biosafe alternative for saliva sample collection. Future large-scale studies are warranted to validate the device’s performance across diverse populations and diagnostic applications.

## Supplementary Information

Supplementary Material 1.

Supplementary Material 2.

Supplementary Material 3.

## Data Availability

The datasets used during the current study are available from the corresponding author on reasonable request.
