# Supplementary material for: Application properties and biosafety enhancement in saliva collection: optimization of a non-blowing medical device
Source: BMC Oral Health. 2026 Jun 11;26:1481. doi: 10.1186/s12903-026-08834-1 (PMC13483494; doi:10.1186/s12903-026-08834-1)

Additional file 1 Results of PCR-HRM for saliva samples (Nos. 001-010) stored for up to 12 months. Images of the normalized reporter after PCR-HRM with various devices (limit test). Commercial device, detection kit was used for PCR-HRM analysis with 70 ng DNA per reaction. New device, point mutation was identified with a minimum limit of 21 ng of DNA per reaction.Positive reference materials included Sample 004 (heterozygous, AG) and Sample 010 (homozygous, GG).

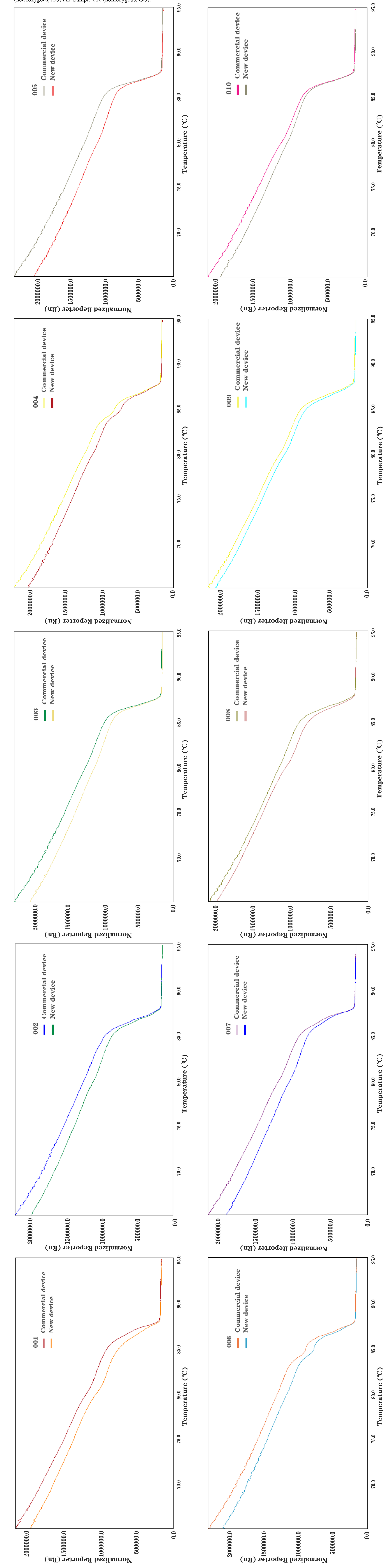

Supplement: Supplementary file 1 [file 12903_2026_8834_MOESM1_ESM.pdf]
