# Supplementary material for: Application properties and biosafety enhancement in saliva collection: optimization of a non-blowing medical device
Source: BMC Oral Health. 2026 Jun 11;26:1481. doi: 10.1186/s12903-026-08834-1 (PMC13483494; doi:10.1186/s12903-026-08834-1)
Supplement: Supplementary file 2 [file 12903_2026_8834_MOESM3_ESM.pdf]

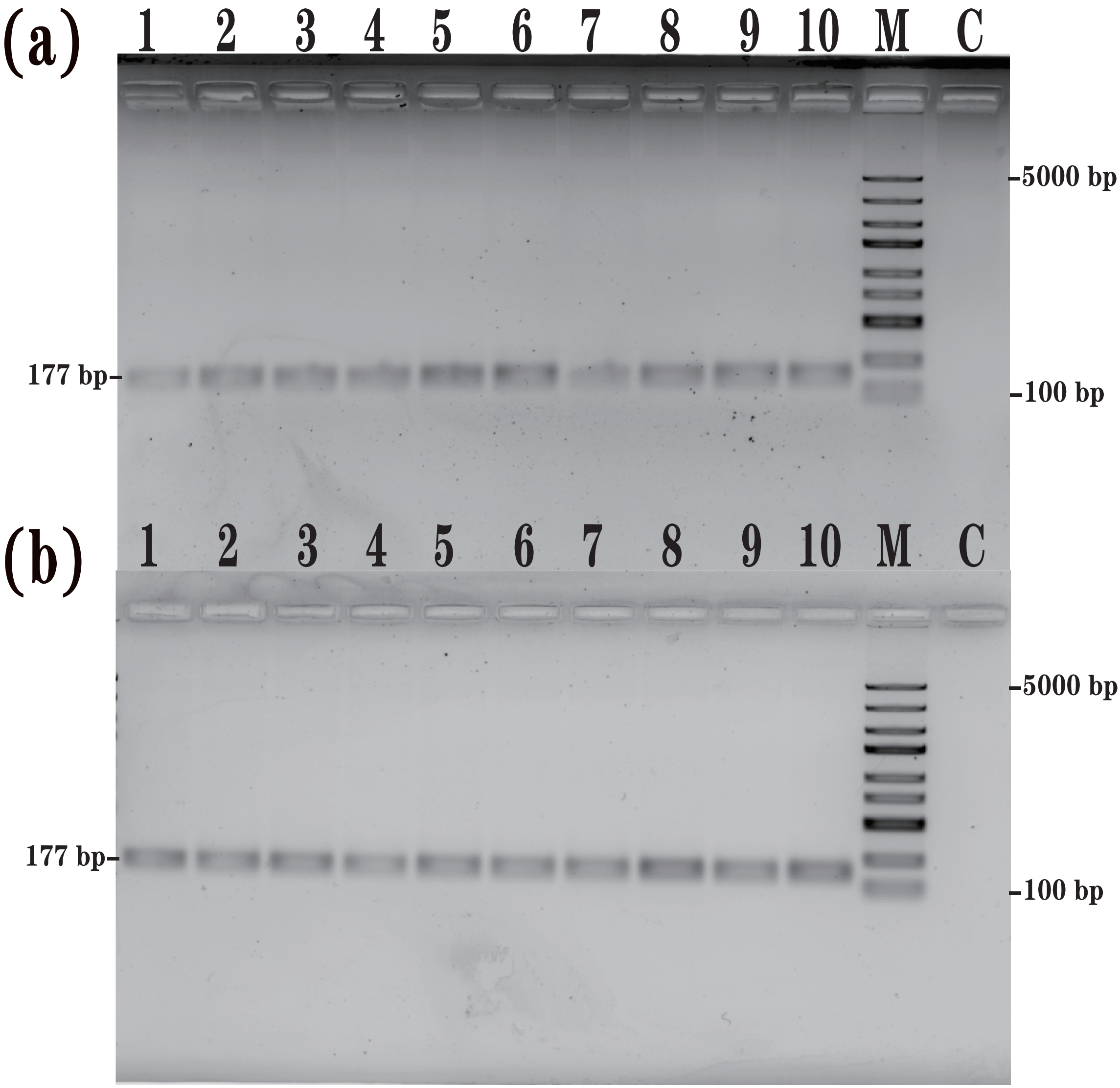

Additional file 2 POU5F1 fragment (177 bp) was amplified by PCR. DNA template derived from the new device (room temperature, a) and commercial device (b). *Lanes* 1-10, electrophoretic bands for saliva samples (Nos. 001-010); *Lane* M, DL5,000 DNA marker; *Lane* C, negative control.
